# Investigating the molecular mechanisms of Tamoxifen on the EMT pathway among patients with breast cancer

**DOI:** 10.25122/jml-2022-0085

**Published:** 2022-06

**Authors:** Mohammadhossein Mirzaei, Seyed Amir Sheikholeslami, Arsalan Jalili, Ahmad Bereimipour, Sheida Sharbati, Vahid Kaveh, Sina Salari

**Affiliations:** 1 Visveswarapura Institute of Pharmaceutical Sciences, Rajiv Gandhi University of Health Sciences, Bangalore, India; 2 Hematology and Oncology Department, Imam Hossein Hospital, Shahid Beheshti University of Medical Sciences, Tehran, Iran; 3 Hematopoietic Stem Cell Research Center, Shahid Beheshti University of Medical Sciences, Tehran, Iran; 4 Department of Stem Cells and Developmental Biology, Cell Science Research Center, Royan Institute for Stem Cell Biology and Technology, ACECR, Tehran, Iran; 5 Department of Basic Medical Sciences, Parvaz Research Ideas Supporter Institute, Tehran, Iran; 6 Faculty of Sciences and Advanced Technologies in Biology, University of Science and Culture, Tehran, Iran; 7 Department of Pharmaceutics, School of Pharmacy, Shahid Beheshti University of Medical Sciences, Tehran, Iran; 8 Hematology and Oncology Department, Iran University of Medical Sciences, Tehran, Iran; 9 Hematology and Oncology Department, Taleghani Hospital, Shahid Beheshti University of Medical Sciences, Tehran, Iran

**Keywords:** Tamoxifen, breast cancer, bioinformatics analysis, EMT, metastasis

## Abstract

Tamoxifen is one of the most used drugs for breast cancer. This study aimed to investigate the effect of the Tamoxifen mechanism on the epithelial-mesenchymal transition (EMT) pathway among breast cancer patients due to its resistance to breast cancer cells. We selected the appropriate datasets from the GEO database using continuous and integrated bioinformatics analysis. We examined the signaling pathways, gene ontology, and protein association of genes after classifying the gene expression profile. Finally, we confirmed the candidate genes using the GEPIA database. Two groups were defined for gene expression profiles. The first group in which the expression profile of genes increased after Tamoxifen was evaluated using the expression profile of genes that decreased in the EMT pathway. The second group was the opposite of the first group. 253 genes in the first group and 302 genes in the second group were shared. The genes in the first group were involved in various pathways of cell death, focal adhesion, and cellular aging. The second group was more involved in different phases of the cell cycle. Finally, MYLK, SOCS3, and STAT5B proteins from the first group and BIRC5, PLK1, and RAPGAP1 proteins from the second group were selected as candidate proteins in connection with the effect of Tamoxifen on the EMT pathway. We evaluated Tamoxifen's effect on the EMT pathway more accurately. However, for a closer look at Tamoxifen, more studies need to be done on target genes and proteins to clarify their role.

## INTRODUCTION

Breast cancer is one of the most common cancers in women, and every year, many women get breast cancer in different stages [1]. In terms of mortality, it is the second most common cancer among women. Despite the many treatments and various drugs used to manage breast cancer patients, breast cancer cell metastasis to other body organs, especially the lungs, still causes many deaths [2].

One of the critical signaling pathways that can enhance the aggressiveness of cancer cells and increase the migration of cells to other body organs is the epithelial-mesenchymal transition (EMT) pathway [3]. Inducing the properties of mesenchymal cells into epithelial cells and the properties of stem cells in general causes the progression and recurrence of breast cancer [4]. Therefore, studying the effects of drugs on the EMT pathway in breast cancer can be considered valuable for targeted and improved therapies to increase the effectiveness of drugs.

Tamoxifen is an anti-estrogen drug that binds to the estrogen receptor in the endoplasmic reticulum and prevents the stimulation of estrogen expression in breast cells [5]. This drug played an essential role in treating breast cancer patients and increased patients' survival by an average of 15 years [6]. Nevertheless, drug resistance of cancer cells has increased in recent years, and drug performance has decreased [7, 8]. Therefore, a closer look at the mechanism of action of Tamoxifen in breast cancer cells and on the vital pathway of cell invasion, namely EMT, can provide appropriate therapies or combinations of adjuvant drugs along with Tamoxifen for better efficacy.

For this purpose, modern methods such as bioinformatics analysis can significantly help find signaling pathways and study the nature of genes. On the other hand, this science can predict the proteins, genes, and regulatory elements involved in cancer [9]. Finally, better strategies can be adopted to evaluate drug mechanisms. In this study, we used the datasets available in reputable databases to assess the gene expression profile of breast cancer patients receiving Tamoxifen and those whose epithelial and mesenchymal cells were isolated to examine the EMT pathway and, eventually, the genes and products. We evaluated more precisely the protein that Tamoxifen can target.

## MATERIAL AND METHODS

### Selection of the appropriate database

In this study, GSE147271 and GSE13915 datasets were selected from the GEO database. The GSE147271 dataset consisted of 121 samples, 60 samples from breast cancer patients before Tamoxifen, and 61 samples after Tamoxifen administration, grouped with their gene expression profiles. The platform of this database is GPL28292 Agendia human array. The GSE13915 dataset consisted of 20 samples containing epithelial and mesenchymal cell samples obtained from patients with breast cancer. The platform of this database is also GPL7785 SMD Print_1354.

### Preparation of data for bioinformatics analysis

We evaluated the datasets using the GEO2R tool. Then we saved all the gene profiles between the groups and uploaded them to an Excel file. We then categorized the genes that were up and downregulated. At this stage, the P-value<0.05 was considered.

### Verifying signaling pathways

To examine the Tamoxifen mechanism in the EMT pathway, we evaluated the expression profile of genes that increased after Tamoxifen with genes that decreased in the EMT pathway. We did the same for the expression profiles of the genes that decreased expression after Tamoxifen and the genes that increased expression in the Venn version 2 diagram's EMT pathway. We then loaded the list of common genes from the two approaches separately into the Enrichr database and separated the signal path associated with these genes using the KEGG library. At this stage, the P-value<0.05 was considered.

### Investigation of gene ontology

We reloaded the two Venn diagrams containing common genes to evaluate biological processes and molecular functions separately in the Enrichr database. We separated them from the ontology, biological processes, and molecular functions. The GOnet database was used to map the network of molecular functions between genes. The P-value<0.05 was considered.

### Protein network

In this part of the study, to investigate the relationship between protein products produced by genes and to examine more closely to select the appropriate genes, we uploaded a list of common genes from Venn diagrams to the STRING database and evaluated the relationship between proteins.

### Evaluation of candidate genes with cancer data

After evaluating all the analyzes of the previous stages and especially examining the network between proteins, genes with more connections and more important connections with other protein products that showed a more prominent role in the network were selected and expressed using the GEPIA database. Attendance at various stages of breast cancer as well as survival was assessed.

## RESULTS

### In the EMT pathway, cell adhesion-dependent signaling pathways, cell death types, and cell cycle were targeted by Tamoxifen

**Figure 1:**
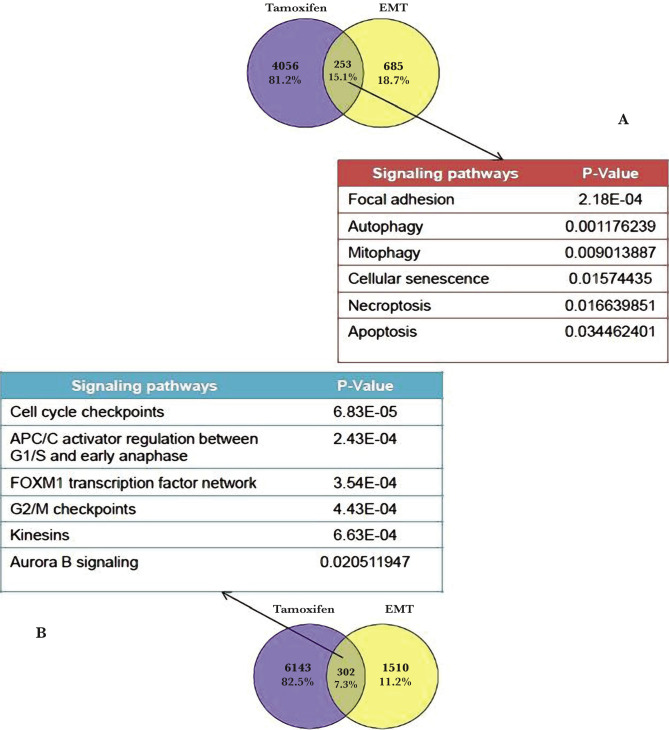
A – The similarities between the expression profiles of genes that were overexpressed by Tamoxifen and decreased in the EMT pathway are isolated, and their signaling pathway is identified. B – The similarity between the expression profiles of genes that reduced expression by Tamoxifen and genes that increased expression in the EMT pathway were isolated, and their signal pathway was evaluated.

After isolating the gene expression profiles of breast cancer patients who received Tamoxifen and another dataset from which the epithelial and mesenchymal cells of breast cancer patients were isolated, there were 253 genes highly expressed in Tamoxifen that were low in the EMT pathway. On the other hand, 302 genes in Tamoxifen had a low expression, which was high in the EMT pathway. Genes overexpressed by Tamoxifen were involved in the signaling pathways of focal adhesion, autophagy, mitophagy, cellular aging, necrosis, and apoptosis. Genes reduced by Tamoxifen expression were involved in the signaling pathways of cell cycle checkpoints, kinesins, FOXM1, G2/M checkpoints, APC, and aurora B. In general, it can be said that high-expression genes in Tamoxifen were more involved in inducing cell death and cell aging. In contrast, low-expression genes in Tamoxifen were more active in different cell cycle phases associated with the EMT pathway (Figure 1 A, B).

### Evaluation of gene ontology targeted by Tamoxifen in the EMT pathway

**Figure 2:**
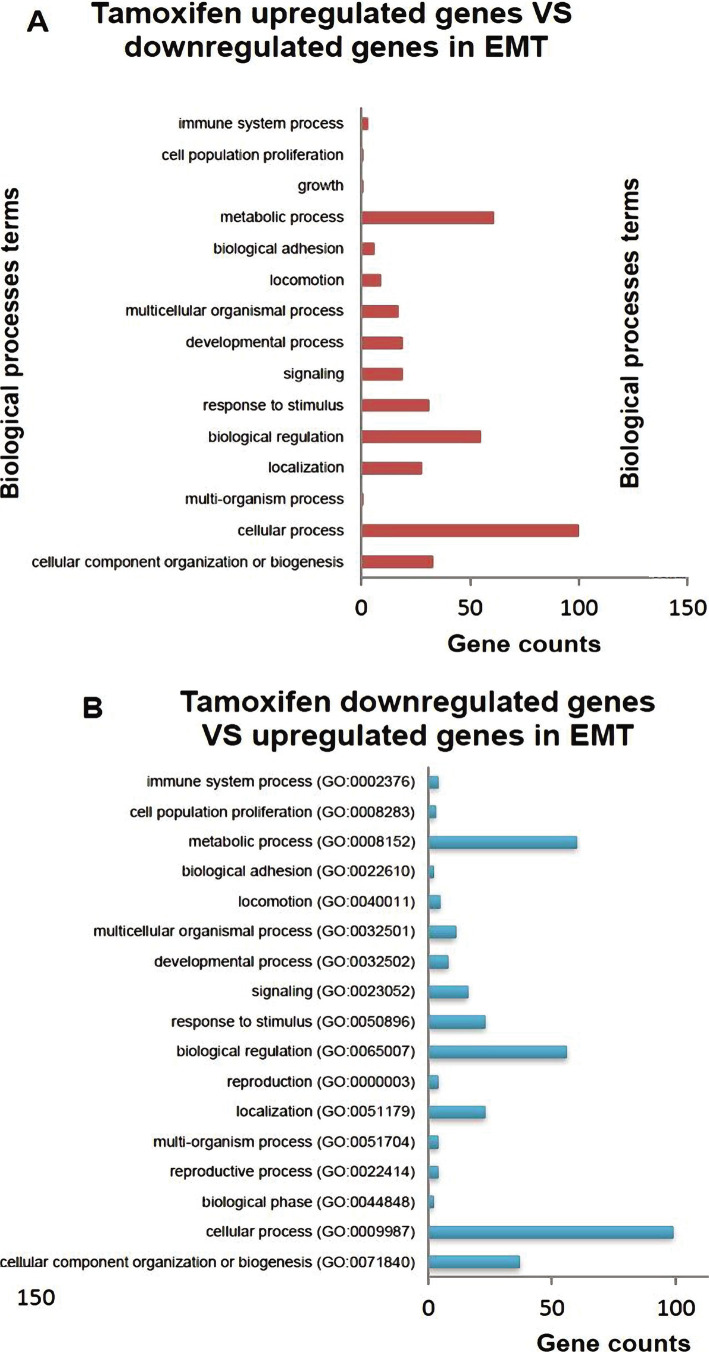
A and B – The study of biological processes between the two groups defined in the research and the study of genes involved in each biological process.

In this part of the study, the ontology of genes in biological processes and molecular functions were examined. Genes overexpressed by Tamoxifen played a key role in cell adhesion pathways. However, genes that showed reduced expression by Tamoxifen showed more activity in their metabolic pathways (Figure 2 A, B).

### Investigation of the association between protein products tampered of EMT by Tamoxifen

**Figure 3:**
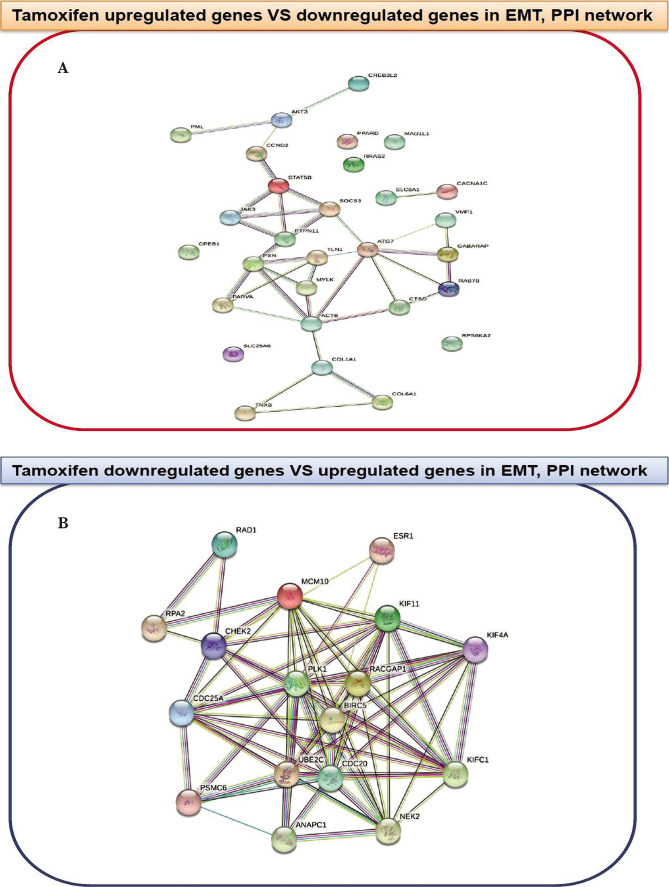
A – The protein network is plotted for overexpressed genes by Tamoxifen and B – for genes with low expression by Tamoxifen. In this network, node degree and betweenness scores were evaluated, and BIRC5, PLK1, RAPGAP1, MYLK, SOCS3, and STAT5B proteins were selected for investigation in human breast cancer samples.

As shown in Figure 3 (A, B), the network between proteins depicts genes that had increased and decreased expression after Tamoxifen. MYLK, SOCS3, and STAT5B proteins overexpressed by Tamoxifen, and BIRC5, PLK1, and RAPGAP1 downregulated by Tamoxifen with a significant relationship in the protein network were selected.

### Verification of key genes in human breast cancer samples via the Cancer Genome Atlas

**Figure 4:**
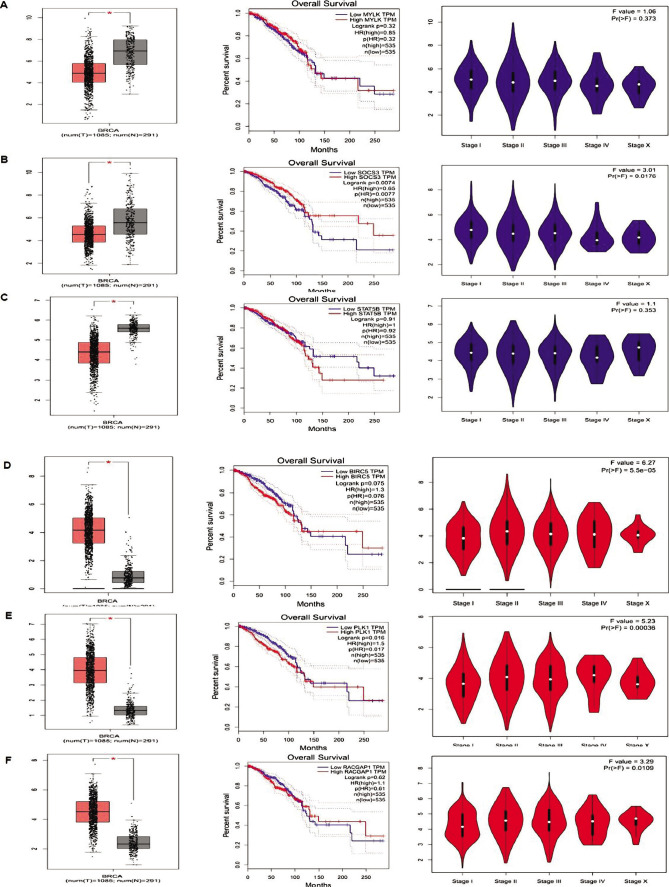
We examined the candidate proteins with the GEPIA database and drew a box plot, stage plot, and survival diagram for 6 candidate proteins. Overall, high and low expression proteins are indicated in stages plots in major stages, and survival plots showed that these proteins reduce survival rate by about 80% in 100 months. A – MYLK; B – SOCS3; C – STAT5B; D – BIRC5; E – PLK1; and F – RACGAP1.

**Figure 5:**
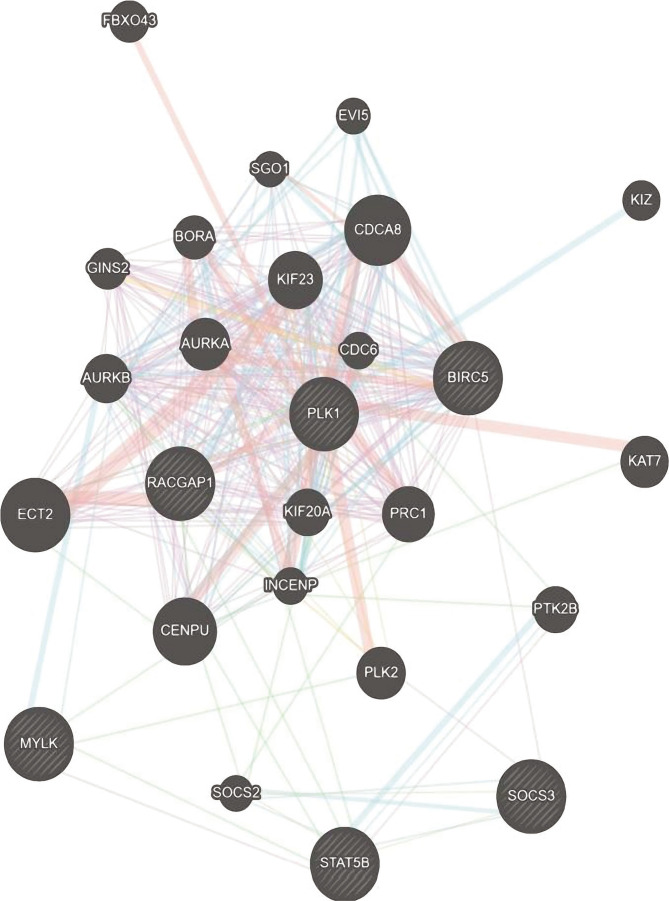
The GeneMANIA database plots the gene network between BIRC5, PLK1, RAPGAP1, MYLK, SOCS3, and STAT5B candidate genes, and the relationship between the genes and their upstream and downstream elements is determined.

Hub genes BIRC5, PLK1, RAPGAP1, MYLK, SOCS3, and STAT5B were investigated in TCGA to verify their roles in BRCA cancer. These genes were surveyed to demonstrate expression differences between normal and BRCA cancer samples, and box plots were drawn. As shown in Figure 4 (A–F), all genes demonstrated statistically significant expression differences. Furthermore, survival analysis of these genes was conducted with the Kaplan-Meier method, and as a result, MYLK, SOCS3, STAT5B, and PLAU genes were obtained with the highest mortality rate for pancreatic adenocarcinoma (PAAD) patients. In other words, patients with a high expression level of these genes showed less survival. We showed expression levels of the genes in various stages of the disease using Violin plots drawn in TCGA. However, all six genes were expressed in all stages. Also, the protein interaction between 6 candidate proteins is shown in Figure 5.

## DISCUSSION

Tamoxifen has been used for many years as a chemotherapy drug for estrogen receptor (ER)-positive women with breast cancer. Tamoxifen, like many chemotherapy drugs, has several side effects for patients [10]. Despite the long-term use of Tamoxifen to treat patients with breast cancer, we have faced drug resistance of breast cancer cells in recent years. This phenomenon led many researchers to study the resistance of Tamoxifen in breast cancer and conduct various studies in this field. However, the point that can be made is that each drug with its unique structure can have a different action mechanism. Therefore, this study investigated the nature of Tamoxifen's molecular mechanism in one of the important pathways involved in the invasion and migration of cancer cells to other body organs, namely EMT, which yielded impressive results.

As mentioned, studies in recent years focused on pathways that inhibit the action of Tamoxifen on breast cancer cells. A cohort study by Liang et al., performed on 4,142 patients, showed that MCAM/CD146 in the EMT pathway increased the inhibition of Tamoxifen's effect on breast cancer cells by activating the Akt signaling pathway [11]. The study by Bui et al. showed that the NOTCH4/STAT3 signaling pathway plays a vital role in the EMT pathway to inhibit Tamoxifen's effect. NOTCH1 and NOTCH4 play a key role in the EMT pathway to increase the migration and invasion of cancer cells to other body organs such as the lungs and especially the liver. NOTCH4 showed a more critical role than NOTCH1 in facilitating cell invasion. NOTCH4, on the other hand, enhances the STAT3 pathway and can be effective in amplifying breast cancer cells and reducing the effect of Tamoxifen [12]. Another study by Yu et al. showed that regulatory elements such as miR-200b/c could affect essential genes in the EMT pathway, such as ZEB1/2 and Vimentin. Thus, low expression of miR-200b/c induces increased expression of ZEB1/2 and vimentin, which is effective in increasing metastasis and invasion of breast cancer cells, and on the other hand, inhibits the function of Tamoxifen [13].

Bioinformatics analyses in this study showed that Tamoxifen targeted and induced various types of cell death in breast cancer cells, such as autophagy, mitophagy, necrosis, and apoptosis. On the other hand, it suppresses genes that are involved in different phases of the cell cycle. In this study, to better investigate the molecular mechanism of Tamoxifen's effect on the EMT pathway, we shared genes with increased expression by Tamoxifen with genes with decreased expression by the EMT pathway. We did the opposite in the same way; we shared the genes reduced by Tamoxifen with the genes overexpressed in the EMT pathway.

One of the most important chemotherapy drug goals is to induce cell death and inhibit the cell cycle. Several studies reviewed different approaches to tamoxifen and breast cancer cells. Tamoxifen can induce cell death in breast cancer cells by inhibiting epidermal growth factor receptors [14] or activating caspase-9 [15]. Various studies were also performed on cell cycle inhibition [16–18].

This study sought to investigate the mechanism of action of Tamoxifen for EMT pathway disorders. For this purpose, after drawing the protein network between the two defined study groups, we selected proteins that played a more important role in the network and established more relationships with other proteins. Then, to finalize the genes and protein products in question, we evaluated the crucial genes in the GEPIA database and finally confirmed MYLK, SOCS3, STAT5B, BIRC5, PLK1, and RAPGAP1 proteins significantly in this cancer database. MYLK, SOCS3, and STAT5B proteins were highly expressed in the EMT pathway, which decreased their expression by Tamoxifen. On the other hand, BIRC5, PLK1, and RAPGAP1 proteins reduced their expression in the EMT pathway, which was increased by Tamoxifen.

Limited studies were performed on MYLK in breast cancer. Myosin light chain kinase is one of the most important genes in facilitating cytoskeletal filaments, which plays a key role in creating a contractile ring in cell division. A recent study by Paul et al. examining gene expression profile in breast cancer cell metastasis showed that the MYLK gene is disrupted and mutated in advanced stages of the disease [19]. However, various other studies related to MYLK and other cancers such as bladder [20], cervical [21], prostate [22], and hepatocarcinoma [23] showed that MYLK increases the invasion and migration of cancer cells to other organs in the body.

Suppressor cytokine signal 3 (SOCS3) is one of the most important tumor suppressors in inhibiting breast cancer cells by regulating inflammatory factors, strengthening the immune system, and invoking immune cells. Kim et al.'s study of breast cancer showed that when tumor suppressors such as P53 and PTEN are disrupted, they pave the way for developing and invading breast cancer cells. At this time, SOCS3 enters into action and, to prevent over-invasion of cancer cells, in cooperation with Interleukin 6, provides conditions for strengthening the immune system and inflammatory responses [24]. A study by Xu et al. among breast cancer patients exposed to cancer cells invading other body organs showed that the miR-203a/SOCS3 axis plays a significant regulatory role in the progression of breast cancer invasion and metastasis [25]. In contrast, Zhang et al. showed that suppression of SOCS3 by EZH2 intensified the invasion and migration of breast cancer cells [26].

Among the various studies performed on STAT5B, most identified STAT5B as a downstream or upstream element for regulating signaling pathways such as cSrc and hypoxia. A survey by Peck et al. on 936 patients with breast cancer showed that low STAT5A expression was associated with metastasis progression in breast cancer cells. However, decreased STAT5B expression was not directly associated with breast cancer invasion. In this study, bioinformatics analysis showed that low STAT5B expression was significantly present in patients with breast cancer and could be involved as a key regulatory element in facilitating cancer cells' progression [27]. These three examined proteins show that their low expression causes pathogenesis and increased invasion of breast cancer cells that were increased by Tamoxifen. It can be said that Tamoxifen targets these three proteins well in the EMT pathway.

One of the most critical proteins in the development of various cancers is BIRC5. This protein can play a vital role in the invasion and metastasis of breast cancer cells by inhibiting apoptosis and increasing cell division. The study by Wang et al. showed that increased expression of BIRC5 and LASP1 was involved in breast cancer metastasis to other organs [28]. Furthermore, a recent study by Dai et al. found that increased BIRC5 expression was effective in breast cancer progression [29].

Another protein that plays an important role in the cell cycle and its increased expression in many cancers and is considered the cause of invasion and metastasis is PLK1. A study by Yao et al. showed that increased PLK1 expression was directly associated with metastasis in breast cancer [30]. Another intriguing study by Ueda et al. showed that knocking down PLK1 inhibited the cell cycle in the G2/M phase, increasing the induction of apoptosis in breast cancer cells. PLK1 in interphase and mitosis has also been shown to play a significant role in promoting cell division in breast cancer cells [31] (Figures 4 and 5).

RACGAP1 is another protein that plays a major role in cell division, cell motility, cytoskeletal organization, and cell migration. Few studies have been performed on RACGAP1 and breast cancer. However, several studies showed increased RACGAP1 expression as a marker of cell division in breast cancer and a diagnostic marker in the early stages [32, 33].

However, the study of the signaling pathways associated with these proteins still requires further extensive studies in the future to indicate what other molecular pathways these proteins overshadow to facilitate metastasis, increase cell division, and invade other organs in the body. The three proteins RACGAP1, PLK1, and BIRC5, play a very important role in cell division, which are also very important for increasing the quality of the EMT pathway. In this study, it was shown that these three proteins are reduced by Tamoxifen.

## CONCLUSION

Finally, it can be said that studying the molecular mechanisms of Tamoxifen, which is used as a common drug for the treatment of breast cancer and has recently encountered drug resistance, can provide better solutions as well as the use of adjuvant drugs to combat the invasion of breast cancer cells.

## Data Availability

All data generated or analyzed during this study are included in this published article.

## Personal thanks

We thank all colleagues in the core facilities for their support.
